# Bulk TiB_2_-Based Ceramic Composites with Improved Mechanical Property Using Fe–Ni–Ti–Al as a Sintering Aid

**DOI:** 10.3390/ma7107105

**Published:** 2014-10-21

**Authors:** Chao Yang, Hao Guo, Daguang Mo, Shengguan Qu, Xiaoqiang Li, Weiwen Zhang, Laichang Zhang

**Affiliations:** 1 National Engineering Research Center of Near-net-shape Forming for Metallic Materials, South China University of Technology, Guangzhou 510640, China; E-Mails: guo.hao@mail.scut.edu.cn (H.G.); daguang.mo@gmail.com (D.M.); qusg@scut.edu.cn (S.Q.); lixq@scut.edu.cn (X.L.); mewzhang@scut.edu.cn (W.Z.); 2 School of Engineering, Edith Cowan University, 270 Joondalup Drive, Joondalup, Perth, WA 6027, Australia; E-Mail: l.zhang@ecu.edu.au

**Keywords:** titanium diboride, metallic additives, spark plasma sintering, microstructure, mechanical properties

## Abstract

The densification behavior, microstructure and mechanical properties of bulk TiB_2_-based ceramic composites, fabricated using the spark plasma sintering (SPS) technique with elements of (Fe–Ni–Ti–Al) sinter-aid were investigated. Comparing the change of shrinkage displacement of pure TiB_2_ and TiB_2_–5 wt% (Fe–Ni–Ti–Al), the addition of elements Fe–Ni–Ti–Al into TiB_2_ can facilitate sintering of the TiB_2_ ceramics. As the sintering temperature exceeds 1300 °C, the relative density does not significantly change. Alumina particles and austenite (Fe–Ni–Ti) metallic binder distributed homogeneously in the grain boundary of TiB_2_ can inhibit the growth of the TiB_2_ grains when the sintering temperature is below 1300 °C. The density and particle size of TiB_2_ greatly influence the mechanical behavior of TiB_2_–5 wt% (Fe–Ni–Ti–Al) composites. The specimen sintered at 1300 has the highest microhardness of 21.1 ± 0.1 GPa with an elastic modulus of 461.4 GPa. The content of secondary borides (M_2_B, being M = Fe, Ni), which are more brittle than TiB_2_ particles, can also influence the fracture toughness. The specimen sintered at 1500 °C has the highest fracture toughness of 6.16 ± 0.30 MPa·m^1/2^ with the smallest M_2_B phase. The results obtained provide insight into fabrication of ceramic composites with improved mechanical property.

## 1. Introduction

Titanium diboride (TiB_2_) has huge potential for applications such as ultra-high-temperature structural materials, cutting tools and lightweight armor materials because of a combination of attractive properties including high melting point, elastic modulus, chemical stability and hardness [1,2]. However, the inherent poor sinterability, brittleness and exaggerated grain growth at high temperature restrict the widely use of monolithic TiB_2_ ceramics in engineering applications [3,4].

Among the methods commonly applied to strengthen and toughen brittle materials, the metal matrix reinforcement method has been studied because it improves not only the toughness but also the sinter ability of TiB_2_ [5]. Research results show that mechanical properties of TiB_2_ ceramics fabricated by the spark plasma sintering (SPS) process with Ti sinter-aid have significantly been improved [6]. Metallic elements, such as iron and nickel, which have lower melting point and better wettability, can be also selected as additives for liquid phase sintering of TiB_2_ [7]. Furthermore, adding controlled additions of Ti and Al into the iron-nickel alloys can prevent the formation of undesirable secondary borides, such as MB, M_2_B and M_23_B_6_, which are even more brittle than TiB_2_ itself [8]. Hence, exploring the use of combination between different metallic elements as sintering aid to enhance TiB_2_ ceramics becomes a necessary scientific question. Because of the plastic behavior of different metallic binder phases during fracture, the fracture toughness of the TiB_2_ composites is expected to be increased.

Spark plasma sintering (SPS) is a novel sintering process that allows the densification of ceramics and can be implemented at low temperatures with short sintering times. These characteristics effectively inhibit the grain growth of materials during the sintering process. Furthermore, the spark discharge in the SPS process can easily puncture the oxide film between the surfaces of ceramic particles [9]. Therefore, the SPS process is considered to be suitable for the fabrication of hard-to-sinter materials such as most ceramics and nanocrystalline materials [10]. In the present research, the combination of Fe–Ni–Ti–Al metallic elements was first used as sintering aid to densify TiB_2_ ceramics. Due to respectively different softening temperature range of the elements of Fe, Ni, Ti and Al, the combination of multiple metallic elements will be conducive to promote the re-distribution of powder particle in the whole sintering temperature range, and improve the densification process of TiB_2_ ceramics. Combining with the advantages of SPS technique, the densification behavior, microstructure and mechanical properties of TiB_2_–5 wt% (Fe–Ni–Ti–Al) composites have been investigated. The results obtained may provide some insight into fabrication of high-performance ceramic composites.

## 2. Results and Discussion

### 2.1. Densification Behavior

**Figure 1:**
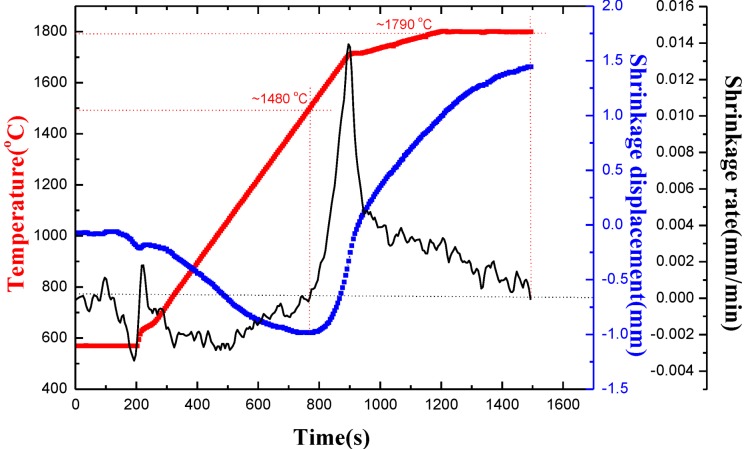
Densification curves for pure TiB_2_ powders sintered at 1800 °C and held for 5 min under 50 MPa.

In the process of sintering, the densification behavior of a sample can be reflected by the displacement of the lower punch, which is automatically stored by the recording system. Figure 1 shows the densification curves for the pure TiB_2_ powders sintered at 1800 °C with a heating rate of 100 °C/min and held for 5 min under 50 MPa. Ignoring some fluctuations of the shrinkage rate below 650 °C due to significant changes in temperature, it is proposed that the densification process begins when the shrinkage rate turns positive and lasts until the shrinkage rate decreases to zero again [11]. For the pure TiB_2_ powders, the densification process begins at approximately 1480 °C and ends at approximately 1790 °C, with approximately unimodal shrinkage rates. The corresponding shrinkage displacement for the pure TiB_2_ powders is about 1.5 mm.

**Figure 2:**
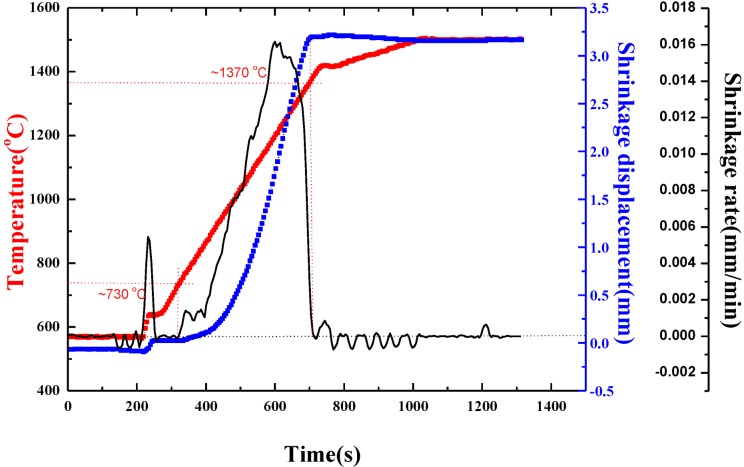
Densification curves for TiB_2_–5 wt% (Fe–Ni–Ti–Al) blended powders sintered at 1500 °C and held for 5 min under 50 MPa.

Figure 2 shows the densification curves for the TiB_2_–5 wt% (Fe–Ni–Ti–Al) blended powders sintered at 1500 °C with a heating rate of 100 °C/min and held for 5 min under 50 MPa. For the blended powders, the starting temperature of the sintering densification process is lowered to approximately 730 °C, and the ending temperature of the densification process is approximately 1370 °C, which is over 400 °C lower than that of the pure TiB_2_ powders. The corresponding shrinkage displacement for the blended powders increases to about 3.2 mm compared to the 1.5 mm shrinkage displacement for the pure TiB_2_ powders. It can be directly concluded that the addition of Fe–Ni–Ti–Al into TiB_2_ powders can not only decrease the densification temperature, but also increase the shrinkage displacement.

The shrinkage-rate curve for the TiB_2_–(Fe–Ni–Ti–Al) blended powders has a similar profile as the pure TiB_2_, which are both unimodal, but the densification mechanism is different. The densification course of pure TiB_2_ is mainly completed by the sintering necks between TiB_2_ particles, which tend to appear at high temperature [9]. For TiB_2_–(Fe–Ni–Ti–Al) blended powders, as the liquid phases increased with rising temperatures below 1370 °C, the shrinkage displacement increased substantially under liquid-sintering, which has a faster transmission speed. The shrinkage displacement of TiB_2_–(Fe–Ni–Ti–Al) is almost twice that for pure TiB_2_ materials. So the TiB_2_–(Fe–Ni–Ti–Al) composites sintered at 1500 °C for 5 min should be more compact than the TiB_2_ materials sintered at 1800 °C for 5 min, which has been proved as following.

**Figure 3:**
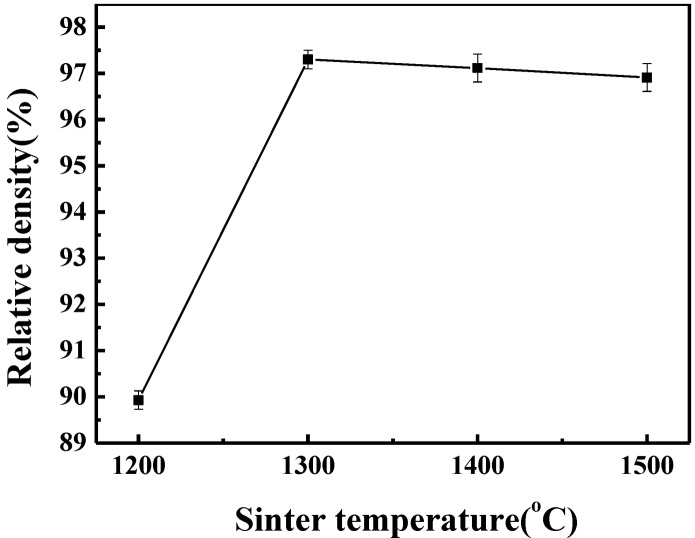
Relative density of the TiB_2_–5 wt% (Fe–Ni–Ti–Al) composites sintered at different temperature.

With the sintering temperature rising from 1200 to 1300 °C, the relative density of the bulk compact increases from 89.9% to 97.3%, as shown in Figure 3. When the sintering temperature exceeds 1300 °C, the relative density does not significantly change, which corresponds to the observation that the densification process of the TiB_2_–(Fe–Ni–Ti–Al) blended powders ends at approximately 1370 °C. Compared with the pure TiB_2_ powders sintered at 1800 °C and held for 5 min, the relative density of the pure TiB_2_ is only 78.6%, which means that the sintering temperature has to be elevated to at least 2000 °C to densify the pure TiB_2_ powders. Thus, the relative density of the TiB_2_ ceramic significantly increased at lower temperatures with 5 wt% (Fe–Ni–Ti–Al) as metallic bonders.

### 2.2. Phase Constitution and Microstructures

**Figure 4:**
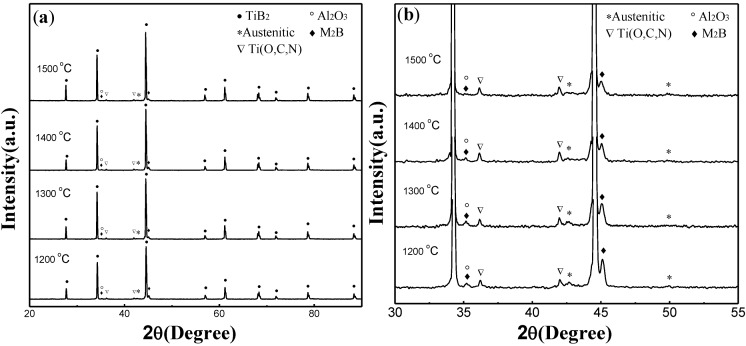
XRD (X-ray diffraction) patterns obtained from the polished surface of TiB_2_–5 wt% (Fe–Ni–Ti–Al) composites sintered at different temperatures: (**a**) full extent and (**b**) selected region.

Figure 4 shows XRD (X-ray diffraction) patterns of the TiB_2_-based ceramic composites fabricated at different temperatures. The diffraction peaks of TiB_2_, (Fe–Ni–Ti) austenite and other phases are presented. The diffraction peak of secondary borides (M_2_B, being M = Fe, Ni) appear in all specimens, which have a negative impact on the fracture toughness of the material. Note that the intensities of the M_2_B peaks decrease as the sintering temperature increases, while the content of the M_2_B phase decreases from 3.8 wt% for a specimen sintered at 1200 °C to 1.2 wt% for a specimen sintered at 1500 °C, calculated from their XRD patterns with the MDI Jade software (Livermore, CA, USA). The content of the brittle phase M_2_B decreases as the sintering temperature increases in the Spark Plasma Sintering (SPS) process because the sintering time increases. In addition, the formation of Al_2_O_3_ phase is attributed to the reaction between aluminum elements with introduced minor oxygen existing in the powders in the sintering process.

**Figure 5:**
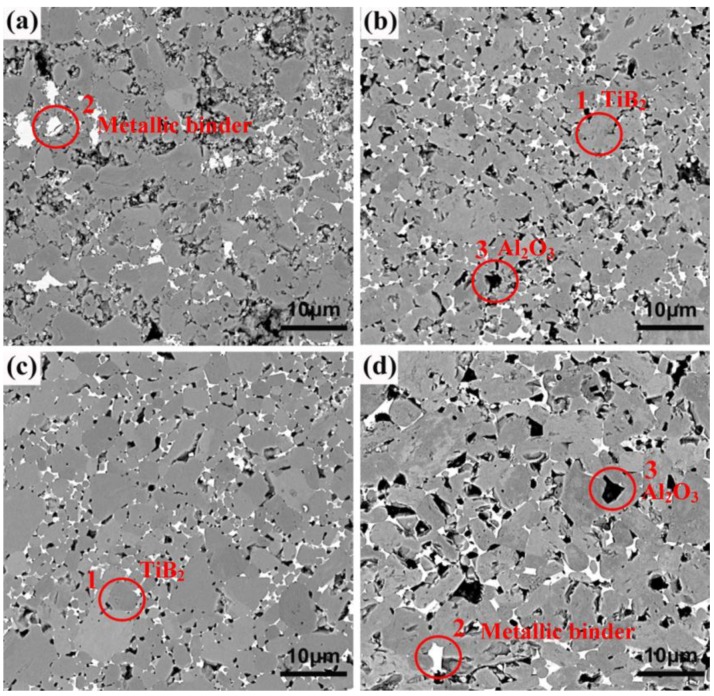
Back-scattered electron microstructures of the TiB_2_–5 wt% (Fe–Ni–Ti–Al) composites sintered at different temperatures: (**a**) 1200 °C; (**b**) 1300 °C; (**c**) 1400 °C; and (**d**) 1500 °C and held for 5 min.

**Figure 6:**
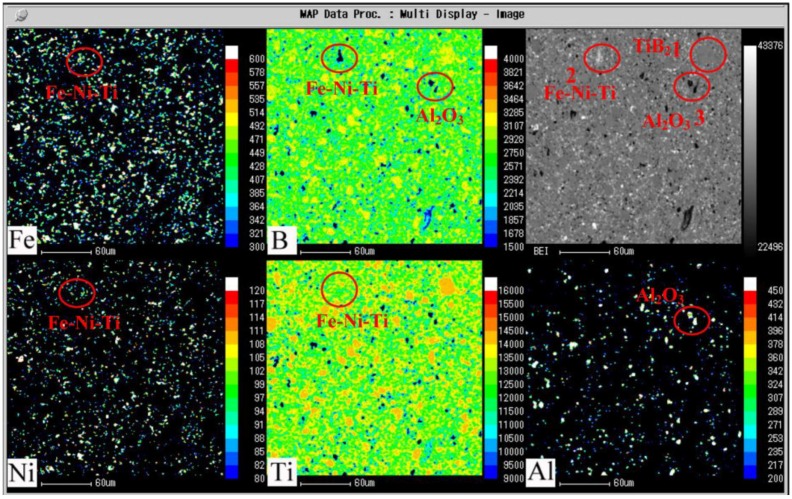
X-ray dot mapping of major elements for the TiB_2_–5 wt% (Fe–Ni–Ti–Al) specimen sintered at 1300 °C and held for 5 min.

The microstructures of the TiB_2_–5 wt% (Fe–Ni–Ti–Al) composites sintered at different temperatures are shown in Figure 5. In all of the figures, there are some black and white areas distributed uniformly in the grey matrix particles. Based on the analysis results of the X-ray dot mapping of major elements for TiB_2_–5 wt% (Fe–Ni–Ti–Al) specimen sintered at 1300 °C (seen in Figure 6): the gray matrix for region-one contains TiB_2_ or Ti (O, C, N ) solid solution, which has little elements except Ti and B; (Fe–Ni–Ti) austenite exists in the white region for region-two, which has almost no B but lots of elements of Fe–Ni–Ti; region-three is the black region, which contain a higher content of aluminum, indicating that the Al_2_O_3_ grains exist in this area.

**Figure 7:**
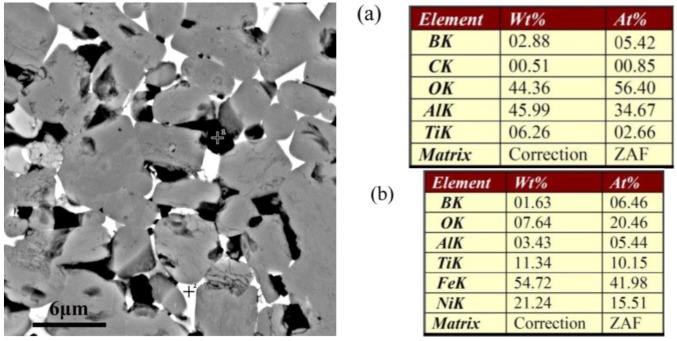
EDS (energy dispersion spectrum) analysis corresponding to the black contrast and white contrast in Figure 5, confirming that the black contrast is mainly alumina, and the white contrast is manly (Fe–Ni–Ti) metallic binder along with secondary borides M_2_B.

Moreover, the energy dispersion spectrum (EDS) analysis corresponding to the black and white contrasts is shown in the Figure 7. The analysis of the black contrast (Figure 7a shows that there is mainly oxygen and aluminum in it, which also have a certain amount of B and Ti due to the influence of the matrix element, confirming that it is mainly alumina. The white area is rich with elements of Fe–Ni–Ti and oxygen (seen in Figure 7b), confirming that it is mainly austenite (Fe–Ni–Ti) metallic binder. In addition, a little boron has also been detected in this area, indicating that secondary borides M_2_B, which resulted from the reaction between TiB_2_ and Fe and Ni, exist along with the (Fe–Ni–Ti) phase.

**Figure 8:**
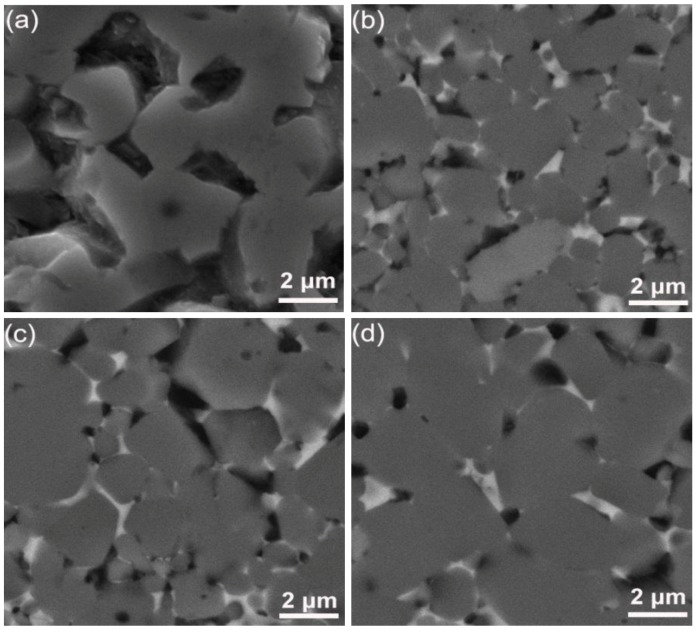
Comparison of SEM (scanning electronic microscope) micrographs of (**a**) the pure TiB_2_ sintered at 1800 °C and the TiB_2_–5 wt% (Fe–Ni–Ti–Al) sintered at different temperatures of (**b**) 1300 °C; (**c**) 1400 °C and (**d**) 1500 °C.

Figure 8 shows the SEM micrographs of the pure TiB_2_ sintered at 1800 °C and the TiB_2_–5 wt% (Fe–Ni–Ti–Al) sintered at different temperatures of 1300, 1400 and 1500 °C. Obviously, the pure TiB_2_ fabricated at 1800 °C has a non-dense microstructure with a relative density of 78.6% and has exaggerated grain growth and some large pores. On the other hand, the microstructure of the TiB_2_–5 wt% (Fe–Ni–Ti–Al) sintered at 1300 °C (Figure 8b) shows that the specimens have multi-scale grains of 1–4 μm and a relative density approaching 97.3%. Considering the starting grain size of TiB_2_ is 1–3 μm, this observation indicates that sintering TiB_2_ ceramic with Fe–Ni–Ti–Al as a sinter-aid by SPS at 1300 °C can not only facilitate densification but also significantly restrain grain growth, which is attributed to the inhibiting growth effect by alumina particles and the (Fe–Ni–Ti) metallic binder (Figure 4, Figure 5, Figure 6, Figure 7 and Figure 8), which exist in the grain boundary of TiB_2_. However, as the sintering temperature exceeds 1300 °C, high temperature softened the alumina particles and the (Fe–Ni–Ti) metallic binder, the inhibiting growth effect seems to be weakened, the grain size of TiB_2_ increases to 2–6 μm when the sintering temperature rises to 1500 °C (Figure 8d), and that will have an adverse impact on mechanical property.

### 2.3. Mechanical Properties

**Figure 9:**
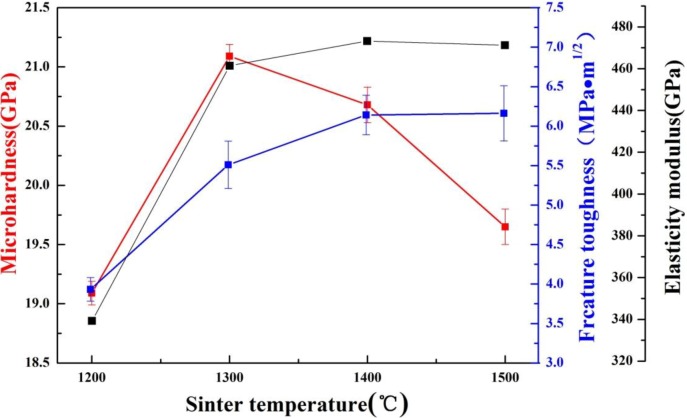
Influence of sintering temperature on the mechanical properties of the TiB_2_-based ceramic composites with 5 wt% (Fe–Ni–Ti–Al) sinter-aid.

The influence of sintering temperature on the mechanical properties of the TiB_2_-based ceramic composites fabricated with 5 wt% (Fe–Ni–Ti–Al) sinter-aid is shown in Figure 9. The microhardness increases from 19.1 ± 0.2 to 21.1 ± 0.3 GPa as the sintering temperature increases from 1200 to 1300 °C. When the sintering temperature exceeds 1300 °C, the microhardness decreases substantially. In general, microhardness is affected by density and grain size. When the sintering temperature increases from 1300 to 1500 °C, the density does not change, so the grain size becomes the main factor influencing the hardness. The grain sizes of the TiB_2_–5 wt% (Fe–Ni–Ti–Al) specimens sintered at different temperatures are shown in Figure 8. Compared to the grain size of TiB_2_ in a specimen sintered at 1300 °C, the grain size sintered at 1500 °C has growth, which explains why the microhardness decreases when the sintering temperature exceeds 1300 °C.

The elastic modulus of the TiB_2_–5 wt% (Fe–Ni–Ti–Al) ceramics composites do not change after rapidly increasing from 1200 °C to 1300 °C. The elastic modulus of the specimen sintered at 1200 °C is 339.2 GPa. The specimen sintered at 1400 °C has the highest elastic modulus of 473.2 GPa. The elastic modulus change of the TiB_2_–5 wt% (Fe–Ni–Ti–Al) composites sintered at different temperatures is consistent with the increase in relative density in Figure 3, which indicates that density is one of the decisive factors influencing the elastic modulus.

**Figure 10:**
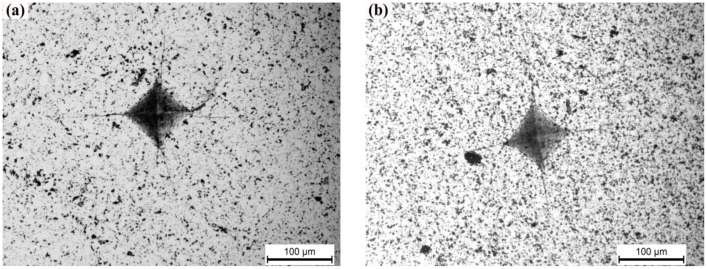
Micrographs of the Vickers hardness indents on the specimen sintered at (**a**) 1200 °C and (**b**) 1300 °C.

Fracture toughness is a material property that resists the propagation of cracks. According to the Equation (1), fracture toughness is affected by the elastic modulus, Vickers hardness, and indentation load and crack lengths. Due to the rapid increase of the relative density, the fracture toughness of the specimens increase from 3.93 ± 0.15 to 5.51 ± 0.30 MPa·m^1/2^ as the sintering temperature rises from 1200 to 1300 °C. As shown in Table 1, the crack length of the specimen sintered at 1200 °C is the largest among the specimens sintered at 1200–1500 °C. Figure 10 shows the two Vickers hardness indents of the specimen sintered at 1200 °C (Figure 10a) and 1300 °C (Figure 10b), with four cracks radiating from each corner of the square indents. It is clear that the average length of the four cracks for the specimen sintered at 1300 °C is shorter than that for the specimen sintered at 1200 °C. According to Equation (1), the fracture toughness is inversely proportional to the crack length, so a shorter crack generally means higher fracture toughness. Furthermore, because of the decline in the microhardness as the sintering temperature exceeds 1300 °C, the fracture toughness of the specimen sintered at 1500 °C further rises to 6.16 ± 0.30 MPa·m^1/2^, higher than that of the monolithic TiB_2_ ceramics with relative density of 97.6%, 5.2 ± 0.4 MPa·m^1/2^ [9]. The improving fracture toughness is due to the effect of grain refinement and the plastic behavior of the metallic binder phase. Meanwhile, the fracture toughness reported here is superior to that of TiB_2_–20 vol% (Fe–Ni) cermets, 5.5 ± 0.8 MPa·m^1/2^ [5], and that of TiB_2_–5 wt% Ti ceramic composites fabricated by the SPS, 5.9 ± 0.3 MPa·m^1/2^ [6], indicating that the effect of combined Fe–Ni–Ti–Al metallic elements as sintering aid to toughen TiB_2_ ceramics is better than that of Fe–Ni and Ti addition. This is because Ti added into the Fe–Ni system can prevent the formation of undesirable secondary borides, and the plastic deformation ability of the austenite (Fe–Ni–Ti) metallic binder is stronger than that of α-Ti. In addition, the fracture toughness of TiB_2_–33wt% (Co–Ti–Al) is only 4.7 ± 0.25 MPa·m^1/2^ [12], indicating that the toughening effect of the Fe–Ni–Ti–Al metallic binder in our case is better than that caused by the Co–Ti–Al system.

**Table 1:** The crack lengths from each corner of the indent to the tip of the corresponding crack of the TiB_2_–5 wt% (Fe–Ni–Ti–Al) composites sintered at different temperatures.

Sintering temperature/°C	C/μm
1200	141.3
1300	123.9
1400	116.2
1500	115.8

On the other hand, the content of secondary borides M_2_B, which are more brittle than TiB_2_ particles, can damage the fracture toughness of the material. As previously described, the content of the brittle phase M_2_B has decreased as the sintering time increases in the SPS (spark plasma sintering) process. The continuously increasing fracture toughness from 1200 to 1500 °C can also be attributed to the decreasing content of the brittle phase M_2_B in the specimens.

**Figure 11:**
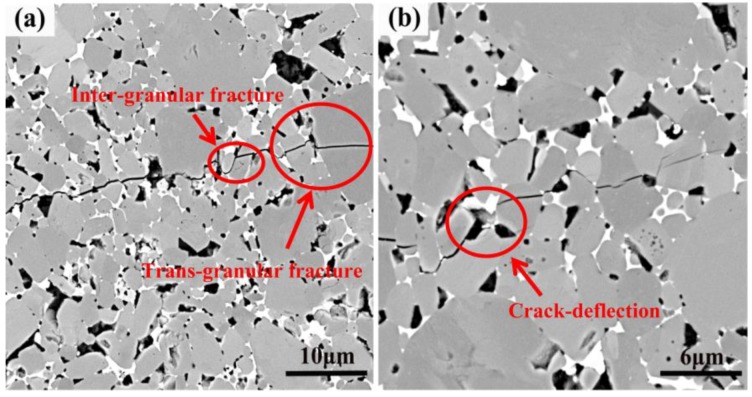
Crack propagation path in the TiB_2_–5 wt% (Fe–Ni–Ti–Al) specimen sintered at 1400 °C for a holding time of 5 min.

In addition, through analysis of the indentation crack of the Vickers hardness test, we can clearly see the crack propagation path of a crack in the TiB_2_–5 wt% (Fe–Ni–Ti–Al) composite. As shown in Figure 11, there are three main fracture modes: inter-granular fracture, trans-granular fracture and crack deflection. Toughening depends mainly on the latter two modes. First, trans-granular fracture occurs in TiB_2_ grains (Figure 11a), which is supposed to increase the fracture toughness because the higher crystal internal energy compared with the crystal boundary energy of the TiB_2_ grains. Second, because of the great difference in thermal expansion coefficient (α) and elastic modulus (*E*) between the (Fe–Ni–Ti) metallic binder and the Al_2_O_3_ particles with TiB_2_ matrix, the crack deflection has appeared at the boundary between them, as shown in Figure 11b. Crack deflection can resist crack growth and consume more energy for the separation of the fracture surfaces [11], so it will further improve the fracture toughness.

## 3. Experimental Procedure 

### 3.1. Starting Powders

The characteristics of the basic powders used to prepare the TiB_2_–metal mixtures are summarized in Table 2. The composition of the metallic binder is Fe–58, Ni–25, Ti–6.3, Al–10.7 (wt%). The basis of composition design is using metallic elements as sintering aid as well as minimizing formation of undesirable secondary borides and oxides. In order to guarantee homogeneous distribution of respective elements, the mixture, composed of 5 wt% (Fe–Ni–Ti–Al) with 95 wt% TiB_2_, was dry milled for 8 h at a milling rotation speed of 150 rpm in a stainless steel vial with stainless steel balls. To minimize the potential contamination of Fe and Cr from the milling balls or vial, the milling was conducted in a low energy mode, in which the milling process paused every 30 min, rested 18 min and finally stopped after 16 cycles. The weight ratio of balls to powder was fixed at 10:1. Furthermore, sieving the milled powders was performed to remove agglomerates, which may lead to poor sinter ability.

**Table 2:** Characteristics of the experimental powders.

Powder	W(C)%	W(O)%	W(N)%	Purity (%)	Mean Particle Size	Shape
TiB_2_	≤0.15	≤0.40	≤0.25	≥99.5	1–3 μm	Spherical
Fe	≤0.05	≤0.2	≤0.05	≥99.5	3–5 μm	Spherical
Ni	≤0.1	≤0.1	≤0.1	≥99.7	2–4 μm	Spherical
Ti	≤0.03	≤0.3	≤0.03	≥99.5	2–4 μm	Irregular
Al	≤0.01	≤0.01	≤0.01	≥99.7	2–4 μm	Irregular

The manufacturer of the experimental powders is Beijing Xingrongyuan Keji Co. Ltd., Beijing, China.

### 3.2. Sintering Parameters

SPS was conducted using a Dr. Sinter Model SPS-825 Spark Plasma Sintering System (Sumitomo Coal Mining Co. Ltd., Osaka, Japan) in a vacuum (≤3 Pa). The mixture powders were poured into a cylindrical graphite die with an inner diameter of Ø20 mm and an outer diameter of Ø50 mm. The temperature was measured by an infrared thermometer though a thermometer hole with the diameter of 2 mm and depth of 7.5 mm located in the center of the die. The selected sintering temperatures were 1200, 1300, 1400 and 1500 °C, with a heating rate of 100 °C/min and an applied pressure of 50 MPa. After soaking the mixtures at a desired temperature for 5 min, the applied current was cut off, and the pressure was released until the specimen was cooled down to room temperature. In addition, a pure TiB_2_ ceramic without doping and ball-milling has been fabricated at 1800 °C using the same heating rate, pressure and dwell time.

### 3.3. Characterization Tests

The microstructure of the fabricated composites was evaluated by high-resolution scanning electron microscopy (SEM, Nova Nano 430, FEI, Hillsboro, OR, USA). Phase identification was conducted by an X-ray diffractometer (XRD, D8 Advance, Bruker Co., Saarbrücken, Germany) using Cu Kα radiation. Based on Archimedes principle, the sintered density was measured using water. The theoretical densities of the specimens were calculated according to the rule of mixtures. The hardness (HV10) was evaluated using a Vickers hardness tester (430SVA, Wilson Wolpert Co. Ltd., Shanghai, China) with a load of 98 N. The fracture toughness (K_IC_) was calculated based on the radial crack length produced by the Vickers (HV10) indentation, according to Anstis’ formula [13]. The reported values are the average of the data obtained from five indentation tests. The elastic properties of the bulk samples were determined using a non-destructive test, *i.e.*, the pulse-echo overlap ultrasonic technique using an ultrasonic detector. The equation used to evaluate *K*_IC_ is:

(1) $$K_{\text{IC}}=16\cdot {\left(\frac{E}{H}\right)}^{1/2}\cdot \frac{P}{C^{3/2}}$$

where *E* is the elastic modulus (GPa); *H* is the Vickers hardness (GPa); *P* is the indentation load (kgN); and *C* is the total crack length (μm) from each corner of the indent to the tip of the corresponding crack.

## 4. Conclusions 

By comparing the change of shrinkage displacement of pure TiB_2_ and TiB_2_–(Fe–Ni–Ti–Al), the addition of metallic elements Fe–Ni–Ti–Al into TiB_2_ can facilitate sintering of the TiB_2_ powder. The densification process of the TiB_2_–(Fe–Ni–Ti–Al) blended powders begins at approximately 730 °C and ends at approximately 1370 °C, which is over 400 °C lower than that of pure TiB_2_. Almost completely densified TiB_2_-based ceramic composites with 5 wt% (Fe–Ni–Ti–Al) sinter-aid was fabricated by the SPS process at a sintering temperature of 1300 °C. As the sintering temperature exceeded 1300 °C, the relative density does not significantly change.

Alumina particles and austenite (Fe–Ni–Ti) metallic binder distributed homogeneously in the grain boundary of TiB_2_ can inhibit the growth of the TiB_2_ grains when the sintering temperature is below 1300 °C. The density and grain size of TiB_2_ greatly influence the mechanical properties of TiB_2_–(Fe–Ni–Ti–Al) composites. As the sintering temperature increases from 1200 to 1300 °C, the microhardness increases from 19.1 ± 0.2 to 21.1 ± 0.3 GPa because the increasing relative density. When the sintering temperature exceeds 1300 °C, the microhardness decreases substantially because the grain size increases, that is due to the high temperature softening of the alumina particles and the (Fe–Ni–Ti) metallic binder weakened the inhibiting growth effect. Change in the elastic modulus keeps pace with the density as the sintering temperature increases from 1200 to 1500 °C. The content of secondary borides (M_2_B, being M = Fe, Ni), which are more brittle than TiB_2_ particles, can also influence the fracture toughness. The specimen sintered at 1500 °C has the highest fracture toughness of 6.16 ± 0.30 MPa·m^1/2^ with the smallest M_2_B phase. Inter-granular fracture, trans-granular fracture and crack bridging are the three main fracture modes, but toughening mainly depends on the latter two modes.
